# Soft prompt-tuning for plant pest and disease classification from colloquial descriptions

**DOI:** 10.3389/fpls.2025.1668642

**Published:** 2025-09-29

**Authors:** Xinlu Liu, Xinbing Li, Yi Zhu

**Affiliations:** ^1^ Engineering Design and Research Institute Co., Ltd, Yangzhou University, Yangzhou, China; ^2^ School of Electrical, Energy and Power Engineering, Yangzhou University, Yangzhou, China; ^3^ School of Information Engineering, Yangzhou University, Yangzhou, China

**Keywords:** plant pests and diseases classification, colloquial descriptions, soft prompt-tuning, verbalizer, natural language processing

## Abstract

The precise identification of plant pests and diseases plays a crucial role in preserving crop health and optimizing agricultural productivity. In practice, however, farmers frequently report symptoms in informal, everyday language. Traditional intelligent farming assistants are built upon domain-specific classification frameworks that depend on formal terminologies and structured symptom inputs, leading to subpar performance when faced with natural, unstructured farmer descriptions. To address this issue, we propose an innovative approach that classifies plant pests and diseases from colloquial symptom reports by leveraging soft prompt-tuning. Initially, we utilize Pretrained Language Models (PLMs) to conduct named entity recognition and retrieve domain-specific knowledge to enrich the input. Notably, this knowledge enrichment process introduces a kind of semantic alignment between the colloquial input and the acquired knowledge, enabling the model to better align informal expressions with formal agricultural concepts. Next, we apply a soft prompt-tuning strategy coupled with an external knowledge enhanced verbalizer for the classification task. The experimental results demonstrate that the proposed method outperforms baseline approaches, including state-of-the-art(SOTA) large language models (LLMs), in classifying plant pests and diseases from informal farmer descriptions. These results highlight the potential of prompt-tuning methods in bridging the gap between informal descriptions and expert knowledge, offering practical implications for the development of more accessible and intelligent agricultural support systems.

## 1 Introduction

Plant pests and diseases are among the most pressing challenges in modern agriculture, threatening crop health, reducing yields, and causing substantial economic losses worldwide (Donatelli et al., 2017; Liu and Wang, 2021). Effective diagnosis and timely intervention are essential to mitigate these threats, particularly in rural and smallholder farming communities where expert support is often limited (Nayagam et al., 2023).

In these real-world agricultural settings, farmers typically report plant symptoms based on their direct observations and personal experiences rather than using standardized scientific terminology (Rodriguez-Garcia et al., 2021). These descriptions are highly colloquial, reflecting local linguistic habits and intuitive interpretations of visible symptoms. For instance, a farmer might describe an infection as “The leaves are yellow and have red dots, with spider-web-like threads on the back”, whereas a technical expert would label the condition as “yellowing with red spider mite infestation.” Similarly, the phrase “The rice has grown white fuzz” might correspond to “powdery mildew” in agronomic terms. Such linguistic mismatches create a substantial barrier between user-reported information and formal agricultural knowledge systems.

Despite the rise of intelligent agricultural assistants powered by natural language processing and image classification technologies, most of these systems are designed around structured, expert-level inputs and rely heavily on terminological consistency (Toscano-Miranda et al., 2022). Current approaches typically require users to select symptoms from predefined categories or input disease names and signs that align closely with entries in agricultural knowledge bases (Wang et al., 2024a). While this design performs adequately in controlled environments or when operated by trained personnel, it fails to accommodate the informal, diverse, and highly variable language used by farmers in natural dialogue (Li and Wang, 2024). As a result, these systems often misclassify or fail to recognize pests and diseases when presented with unstructured, colloquial input. Moreover, the colloquial descriptions are often very short and ambiguous, which exacerbates the challenge of accurate classification. Some recent studies in short text classification have attempted to tackle similar issues of data sparsity and semantic ambiguity by using character-level attention mechanisms combined with feature selection (Zhu et al., 2020), or by leveraging prompt-learning with external knowledge expansion (Zhu et al., 2024). However, these methods generally do not explicitly integrate agricultural domain knowledge nor address the unique linguistic patterns of farmer-reported symptoms, limiting their applicability in this context.

To bridge this gap between colloquial farmer descriptions and formal pest and disease classification, in this paper, we introduce an innovative approach for plant pest and disease classification based on colloquial descriptions by leveraging soft prompt-tuning. Unlike conventional fine-tuning methods that require extensive re-training of model parameters on domain-specific datasets, soft prompt-tuning introduces lightweight, continuous prompt vectors that guide the model’s attention toward relevant linguistic patterns without modifying the core model architecture. Specifically, our method first leverages the AgriBERT based on PLMs for named entity recognition to extract key agricultural entities from the obfuscated text, and the agricultural knowledge graph is introduced to query domain-specific knowledge related to the entities. Then, the user-provided fuzzy description and the retrieved knowledge are concatenated to the soft prompt-tuning model. The external verbalizer further enriches the model’s understanding by mapping informal expressions to corresponding technical terms using structured agricultural knowledge, allowing the model to interpret and classify colloquial symptom descriptions more accurately. By leveraging the generalization capabilities of PLMs and integrating domain knowledge through the constructed verbalizer, our method effectively aligns natural language descriptions with standardized pest and disease categories. Comprehensive experiments conducted on two datasets demonstrate that our method outperforms the SOTA baselines including LLMs. In summary, the primary contributions of our work are outlined below:

1. We identify and address a critical gap in plant pest and disease classification by focusing on the challenge of interpreting colloquial, non-standard symptom descriptions provided in real-world scenarios, which are often overlooked by existing intelligent agricultural systems designed around formal terminology.
2. We introduce an innovative classification approach based on soft prompt-tuning, enhanced with an external knowledge extension verbalizer, which effectively bridges informal linguistic input and domain-specific agricultural knowledge without requiring extra fine-tuning.
3. We construct and evaluate our method on two datasets of real-world, demonstrating superior classification accuracy and robustness compared to the SOTA baselines including LLMs, thus highlighting the practical potential of our method for improving intelligent agricultural diagnostics in real-world scenarios.

## 2 Related work

### 2.1 Plant pests and diseases classification

Plant diseases and pests are significant factors determining both the yield and quality of crops, which can be addressed by means of artificial intelligence (Spence et al., 2020). These diseases and pests represent a form of natural disasters that disrupt the healthy growth of plants, potentially leading to plant mortality throughout the entire development stage, from seed formation to seedling growth (Liu and Wang, 2021).

Traditional approaches to plant pest and disease classification have predominantly relied on manual inspections and specialized knowledge, which are labor-intensive, time-consuming, and prone to human mistakes and biases (Xing and Lee, 2022). With the rise of machine learning and computer vision, automated image-based classification methods have gained widespread attention for their potential to improve efficiency and accuracy (Domingues et al., 2022). For example, Shoaib et al. proposed advanced deep learning models for plant disease detection, highlighting the effectiveness of Convolutional Neural 85 Networks (CNNs) in learning hierarchical features from images (Shoaib et al., 2023). Some classical 86 architectures such as AlexNet, VGGNet, ResNet, and Inception have been employed to classify diseases in various crops, including tomato, rice, maize, and citrus (Sumaya et al., 2024). For instance, Yueteng et al. demonstrated that an improved ResNet architecture enhances recognition accuracy in complex plant disease datasets (Yueteng et al., 2021). Furthermore, traditional machine learning models like Support Vector Machines (SVM), k-Nearest Neighbors (k-NN), and Random Forests have been deployed, often in conjunction with manually extracted features such as color, texture, and shape descriptors. For example, Kale et al. analyzed crop disease detection using these classifiers and found that while effective under certain conditions, they often struggle with generalizability across diverse environmental conditions and are limited when dealing with visually similar symptoms among different diseases (Kale and Shitole, 2021). However, these methods often struggle with generalizability across diverse environmental conditions and are limited when dealing with visually similar symptoms among different diseases.

Recently, to overcome the limitations of single-modal approaches, there have already been some efforts on exploring multi-modal learning frameworks for plant pest and disease classification, which integrate heterogeneous data sources, such as images, textual descriptions, sensor data, and environmental metadata (Yang et al., 2021). This paradigm aims to enhance the robustness and contextual awareness of classification systems (Liu et al., 2025a). For example, Wei et al. proposed a multi-modal transformer architecture for citrus pests and diseases classification, where both image and text features are encoded and aligned through a cross-attention mechanism, enabling improved retrieval and identification performance (Wei et al., 2023). Similarly, Duan et al. introduced a multimodal system combining RGB images, text data, and environmental cues to facilitate pest detection and classification, demonstrating superior performance over image-only models, especially in complex agricultural scenarios (Duan et al., 2023). Wang et al. proposed Agri-LLaVA, an advanced multimodal assistant enriched with domain knowledge, designed specifically for managing 108 agricultural pests and diseases. Agri-LLaVA is trained on an extensive multimodal dataset, containing more than 221 varieties of pests and diseases, amounting to roughly 400,000 data samples. By integrating domain-specific knowledge into its training process, Agri-LLaVA demonstrates superior performance in both multimodal agricultural dialogue and visual comprehension, offering innovative solutions to tackle pest and disease challenges in agriculture (Wang et al., 2024b). These approaches leverage the complementarity of modalities, while images provide morphological cues, textual and contextual data supply semantic and environmental understanding, which proves to be useful for fine-grained and field-based classification tasks.

Recently, to overcome the limitations of single-modal approaches, there have already been some efforts on exploring multi-modal learning frameworks for plant pest and disease classification, which integrate heterogeneous data sources, such as images, textual descriptions, sensor data, and environmental metadata (Yang et al., 2021). This paradigm aims to enhance the robustness and contextual awareness of classification systems (Liu et al., 2025a). For example, Wei et al. proposed a multi-modal transformer architecture for citrus pests and diseases classification, where both image and text features are encoded and aligned through a cross-attention mechanism, enabling improved retrieval and identification performance (Wei et al., 2023). Similarly, Duan et al. introduced a multimodal system combining RGB images, text data, and environmental cues to facilitate pest detection and classification, demonstrating superior performance over image-only models, especially in complex agricultural scenarios (Duan et al., 2023). Wang et al. proposed Agri-LLaVA, an advanced multimodal assistant enriched with domain knowledge, designed specifically for managing 127 agricultural pests and diseases. Agri-LLaVA is trained on an extensive multimodal dataset, containing more than 221 varieties of pests and diseases, amounting to roughly 400,000 data samples. By integrating domain-specific knowledge into its training process, Agri-LLaVA demonstrates superior performance in both multimodal agricultural dialogue and visual comprehension, offering innovative solutions to tackle pest and disease challenges in agriculture (Wang et al., 2024b). In addition, Zhao et al. introduced PlanText, a gradually masked guidance framework to align image phenotypes with trait descriptions for plant disease texts, further highlighting the potential of integrating visual and textual modalities in plant health analysis (Zhao et al., 2024). Meanwhile, Dong et al. developed PlantPAD, a large-scale image phenomics platform for plant science, which provides high-quality resources for training and validating plant disease classification systems (Dong et al., 2024). These approaches leverage the complementarity of modalities, while images provide morphological cues, textual and contextual data supply semantic and environmental understanding, which proves to be useful for fine-grained and field-based classification tasks.

Although the above-mentioned multi-modal approaches have shown promise in plant pests and diseases classification, most existing methods primarily treat non-visual modalities as auxiliary inputs to enhance image-based features. This image-centric design often overlooks the independent value and discriminative power of other modalities, particularly textual data. In real-world agricultural scenarios, textual descriptions are typically colloquial, non-standard, and context-dependent, posing significant challenges to conventional multi-modal fusion strategies. While some studies have explored robust textual encoding techniques to handle noisy or weakly structured inputs (Zhu et al., 2023), these characteristics nevertheless result in a persistent semantic gap that current models struggle to bridge, thereby limiting their robustness and generalizability. These characteristics lead to a semantic gap that current models struggle to bridge, limiting their robustness and generalizability. To address these limitations, in this paper, we propose a novel approach to improve the model’s capacity to understand and utilize natural language expressions effectively for more accurate and practical plant disease classification.

While multimodal and text-based approaches have achieved progress, existing models still face significant challenges when processing colloquial, non-standard user inputs. Farmers’ symptom descriptions are often short, vague, and expressed in everyday language, which are inconsistent with the professional terminologies used in agricultural knowledge bases. For example, models trained on standardized datasets struggle to align colloquial expressions with technical disease terms, leading to misclassification or failure to recognize symptoms. Moreover, the semantic ambiguity and variability of colloquial text introduce additional noise, weakening the model’s ability to capture fine-grained distinctions across different disease categories. These limitations further underscore the necessity of developing methods that can effectively bridge colloquial language with domain-specific knowledge, which is precisely the problem our study seeks to address.

### 2.2 Prompt-tuning

Prompt-tuning has surfaced as an efficient and effective method for adjusting Pre-trained Language Models (PLMs) to downstream tasks without requiring full model fine-tuning (Liu et al., 2021). This paradigm transferred downstream tasks through cloze-style objectives, which is particularly attractive in resource-constrained settings due to its efficiency and ability to preserve general language knowledge encoded in PLMs. The evolution of prompt-tuning includes both discrete and soft prompt methods. Early work in manual prompt design relied on human intuition to craft natural language prompts that could guide PLMs toward the desired behavior, including relation extraction (Han et al., 2021), knowledge probing (Petroni et al., 2019), and text classification (Hu et al., 2021). For example, Han et al. introduced a prompt-tuning model with rules for many-class classification tasks, encoding prior knowledge into prompt-tuning via logic rules and proposing manually designed sub-prompts to construct task-specific prompts (Han et al., 2021).

However, the manually created prompt proved to be inflexible and suboptimal, leading to the development of automated prompt generation strategies (Li and Liang, 2021). In the soft prompt-tuning, continuous embeddings are served as prompts and optimized while keeping the PLM’s weights frozen. For instance, Shin et al. developed the AUTOPROMPT method for generating prompts across various NLP downstream tasks (Shin et al., 2020). In the method, an auto-prompt consisted of the input sentence and the set of trigger tokens. These tokens remain consistent across all inputs and are determined via a gradient-based search mechanism. Wu et al. proposed an information-theoretic approach that framed soft prompt-tuning as optimizing the mutual information between the prompts and other model parameters (Wu et al., 2023). The technique involved two loss functions to achieve proper prompt initialization and extract relevant task-specific information from downstream tasks. Zhu et al. proposed a soft prompt-tuning method for short text stream classification (Zhu et al., 2025), which builds the verbalizer using internal knowledge rather than retrieving from external knowledge bases, further optimizing it through additional tailored strategies. Considering the advantages of soft prompt in contrast to manually crafted prompts, in this paper, we introduce the soft prompt-tuning method for colloquial descriptions in plant pest and disease classification.

## 3 Methodology

### 3.1 Overall architecture

**Figure 1:**
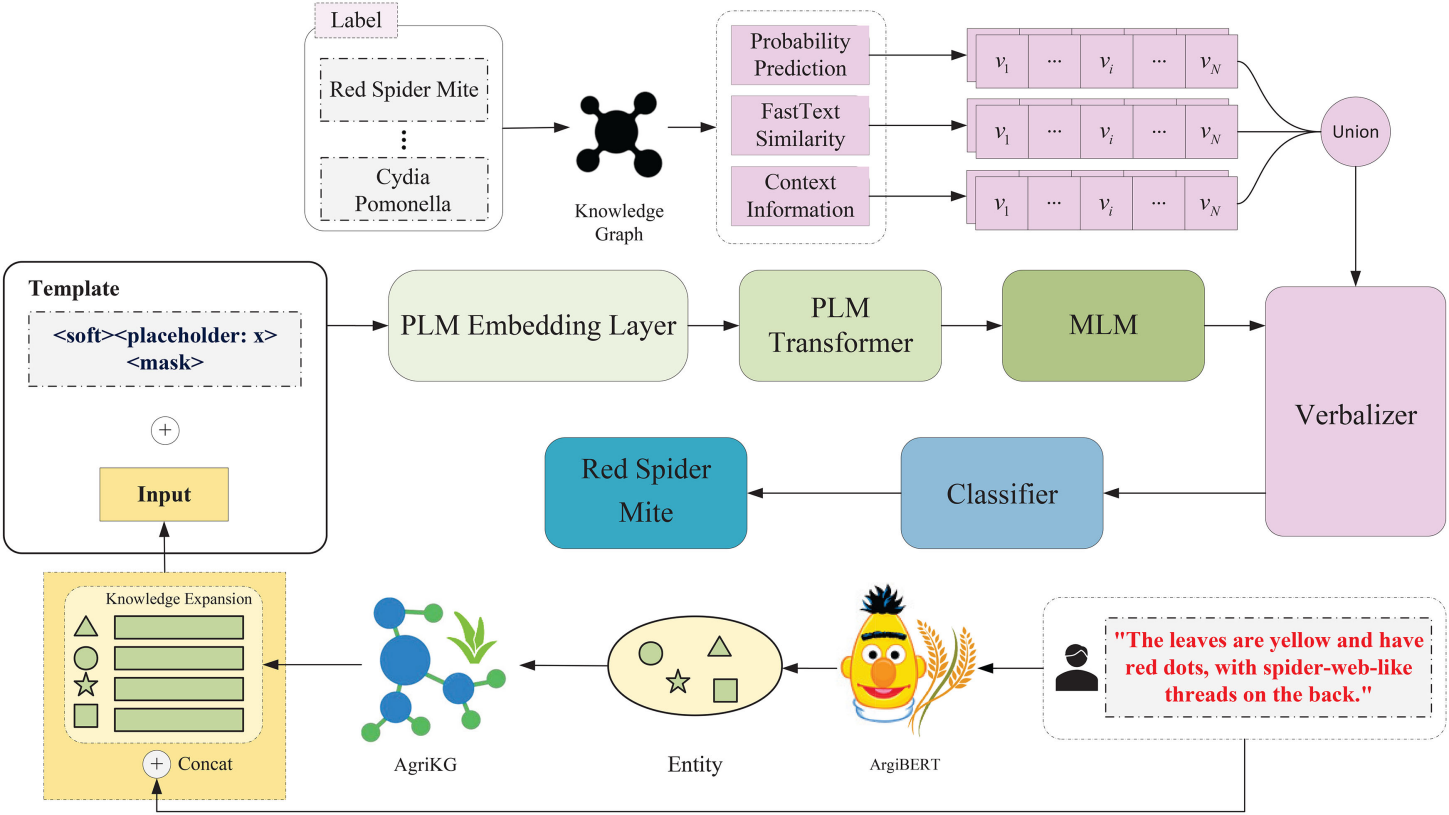
Illustration of the proposed method combining AgriBERT-based entity recognition and AgriKG retrieval, enhanced by prompt learning with soft templates and extended verbalizers for improved pest and disease prediction.

As shown in **Figure 1**, the proposed method first leverages the AgriBERT (Chen et al., 2024) for named entity recognition model to extract key agricultural entities from the obfuscated text, with relevant attribute information retrieved from the AgriKG (Chen et al., 2019) agricultural knowledge graph to effectively supplement domain knowledge. Then, the user-provided fuzzy description and the retrieved knowledge are concatenated to the soft prompt-tuning model. For the prompt-tuning method, the external verbalizer further enriches the model’s understanding by mapping informal expressions to corresponding technical terms using structured agricultural knowledge, allowing the model to interpret and classify colloquial symptom descriptions more accurately. By leveraging the generalization capabilities of PLMs and integrating domain knowledge through the constructed verbalizer, our approach notably enhanced both the precision and interpretability of pest and disease predictions.

### 3.2 Knowledge enhancement

First, we utilize the AgriBERT named entity recognition model, specifically trained for agricultural texts, to extract relevant entities from the input fuzzy agricultural text. This model captures contextual information through a multi-layer bidirectional self-attention mechanism and incorporates a global pointer mechanism for entity localization.

For the input fuzzy text $T_{fuzzy}=\left(t_{1},t_{2},\ldots ,t_{n}\right)$ , where *t_i_* represents the *i*-th word in the text, the AgriBERT model outputs a set of entity labels $E=\left\{e_{1},e_{2},\ldots ,e_{m}\right\}$ , where each entity *e_i_* includes the entity type and its position within the text. However, this positional information is still relatively coarse and cannot guarantee precise boundary detection. We use the global pointer mechanism *P*( $e_{i}$ ) to represent the specific position of entity $e_{i}$ in the text, as described by the following formula (Equation 1):


(1) $$P\left(e_{i}\right)=\left(start_{i},end_{i}\right)$$

where *start_i_* and *end_i_* represent the start and end positions of entity *e_i_*, respectively. Thus, $e_{i}$ provides the semantic label, while *P*( $e_{i}$ ) precisely anchors the boundary, thereby enhancing the model’s robustness in handling overlapping or ambiguous entities.

After extracting the entities, we leverage AgriKG, a publicly available agricultural knowledge graph, to query domain-specific knowledge related to the entities. By querying AgriKG, we obtain the corresponding relevant knowledge *K_i_* for each entity $e_{i}$ , which contains various attributes related to the entity. Let *K_i_* represent the set of knowledge fragments obtained from AgriKG. We organize these knowledge fragments as follows (Equation 2):


(2) $$K_{i}=\left\{k_{1},k_{2},\ldots ,k_{p}\right\}$$

where each knowledge fragment $k_{j}$ provides specific information relevant to entity $e_{i}$ and contains domain related knowledge in agriculture.

Next, we concatenate the user-provided fuzzy description $T_{fuzzy}=\left(t_{1},t_{2},\ldots ,t_{k}\right)$ and the relevant knowledge fragments *K_i_* retrieved from AgriKG. The fuzzy text $T_{fuzzy}$ represents the words in the non-expert language provided by the user. We concatenate the user’s description $T_{fuzzy}$ with the knowledge fragments from AgriKG in the following format (Equation 3):


(3) $$\text{Enhanced Description}=T_{fuzzy}+\left[\text{SEP}\right]+K_{i}$$

where [SEP] is a separator used to distinguish the original description from the knowledge fragments. The concatenated text contains both the user’s non-expert description and the supplemental domain knowledge, thereby enhancing the professionalism and completeness of the text.

To further illustrate this process, we provide two running examples in **Table 1**. Each input is first parsed by AgriBERT-NER to extract entities, then enriched with compact knowledge snippets from AgriKG, and finally concatenated into the enhanced description. As shown in the table, the pipeline effectively aligns colloquial farmer expressions with formal agronomic terminology, leading to accurate classification results.

**Table 1:** Running examples illustrating how the proposed framework processes colloquial farmer descriptions.

Colloquial Input	NER (Entities)	KG Snippets	Enhanced Description
The whole field can look like it’s been burned from far away because so many leaves have turned brown and died.	field, burned, leaves, brown, died	leaf necrosis, burnt appearance, rice blast lesion	Colloquial input followed by [SEP] and knowledge snippets: “leaf necrosis; burnt appearance; rice blast lesion”
The spots on the leaves are a dry, tan, or light brown color and are always surrounded by a bright yellow ring.	spots, leaves, dry, tan/light brown, yellow ring	leaf spot symptom, necrotic lesion, halo chlorosis	Colloquial input followed by [SEP] and knowledge snippets: “leaf spot symptom; necrotic lesion; halo chlorosis”

Each input is first parsed by AgriBERT-NER to extract entities, then enriched with knowledge snippets from the agricultural KG, and finally transformed into an enhanced description.

### 3.3 Soft template construction

We adopt AgriBERT as the backbone PLM. This model is specifically trained on agricultural text tasks, enabling strong capabilities in agricultural terminology recognition and semantic representation. Essentially, AgriBERT follows the BERT architecture, consisting of 12 Transformer encoder layers, each with a 768-dimensional hidden representation and 12 self-attention heads.

In contrast to prompt-tuning methods relying on manually crafted templates, our method utilizes soft templates learned within a continuously optimized prompt space. When integrated with the enhanced description $x_{en}$ described earlier, this approach enables more adaptive text recognition by the model, and can be formulated as (Equation 4):


(4) $$T_{prompt}=\left\{\left[u_{i}\right],\ldots ,x_{en},\ldots ,\left[u_{n}\right],\left[MASK\right]\right\}$$

where *x_en_* represents the enhanced description obtained by concatenating the fuzzy text with the knowledge fragments introduced in Section 3.2, $u_{i}$ denotes the $i^{th}$ learnable token, the prompt T is subsequently passed through the encoder of aPLM to generate hidden states $h_{i},\ldots ,h_{x_{en}},\ldots ,h_{n},h_{\text{MASK}}$ . Accordingly, the soft prompt is formulated as (Equation 5):


(5) $$T_{prompt}=\left\{\left[h_{i}\right],\ldots ,h_{x_{en}},\ldots ,\left[h_{n}],[h_{\text{mask}}\right]\right\}$$

To further enhance the model’s ability to capture temporal and contextual information in non-standard expressions, we integrate a two-layer bidirectional LSTM encoder head into the prompt-learning framework. The input size, hidden size, and embedding size are all set to 768 to maintain consistency between forward and backward information flows. During inter-layer representation transfer, each LSTM layer employs the standard hidden-state propagation mechanism to preserve information progression. The final output is obtained by concatenating the hidden states from both directions, which is then fed into the classifier for prediction. This process can be visualized as (Equation 6):


(6) $$h_{i}^{'}=\left(\vec{h_{i}}\;,\;\overset{\leftarrow }{h_{i}}\right)=\left(\vec{\text{LSTM}}\left(h_{i},\vec{h_{i-1}}\right),\;\overset{\leftarrow }{\text{LSTM}}\left(h_{i},\overset{\leftarrow }{h_{i+1}}\right)\right)$$

where *h_i_* denotes the hidden state input at the *i*-th position of the input sequence, derived from the encoder of AgriBERT. Specifically, it represents the contextualized embedding obtained by feeding the soft prompt together with the textual input into the Transformer architecture. The final fused representation $h_{i}^{'}$ is formed by concatenating the hidden states from the forward and backward directions of the BiLSTM, thereby capturing bidirectional contextual information.

Ultimately, the model improves its performance and output quality by determining the optimal values of variables via the loss function, as illustrated in (Equation 7).


(7) $$h=\text{arg }\underset{h_{i}^{'}}{\min}\;L\left(M\left(x_{en},MASK\right)\right)$$

To adjust the model parameters, we adopt the cross-entropy loss, which quantifies the divergence between predicted outputs and ground-truth labels. The objective function is formulated as (Equation 8):


(8) $$ℒ=-\frac{1}{N}\text{log }p(y^{*}|x_{en})+\alpha |\theta {|}^{2}$$

where N denotes the number of training instances, $y^{*}$ is the gold label, and $|\theta {|}^{2}$ represents the L2 penalty on parameters θ . The penalty term helps alleviate overfitting by restricting parameter magnitudes, and the coefficient *α* balances the impact of regularization in the overall loss.

### 3.4 Verbalizer construction

Prompt-tuning studies (Schick and Schutze, 2020) have shown that aligning label words with their target categories *y* helps reduce the mismatch between textual input and label representation. This process, referred to as automatic label word selection (Gao et al., 2020) or verbalization (Schick et al., 2020), can be formally expressed as (Equation 9):


(9) $$\{v|v_{1},\ldots ,v_{i},\ldots ,v_{N}\}\overset{Mapping}{\to }y$$

where $v_{i}$ represented a word in the verbalizer. In our method, we build the verbalizer by leveraging words extracted from an external knowledge graph. This method expands semantic diversity while promoting greater robustness and generalizability of the verbalizer.

To retrieve mapping words associated with the target categories from a knowledge graph, we employ Related Words^1^ as our external source. This knowledge graph aggregates multiple resources, such as word vectors, ConceptNet (Speer et al., 2017), and WordNet (Pedersen et al., 2004), allowing us to extract an initial set of words v for each category label *y*, thereby constructing the base verbalizer. Given the vast amount of text and the possibility of noise or irrelevant content, we implement three optimization strategies to refine the extracted words. These strategies are designed to capture various facets of the expanded word characteristics, aiming to uncover the underlying intent of the original text. The specific methods are outlined below:

FastText Similarity: A commonly employed method for improving verbalizer construction consists of evaluating the semantic similarity between category labels and their extended label terms. This approach uses the FastText embedding model to generate vector representations and calculate the cosine similarity between category label terms and their expanded equivalents. Let $E_{y}$ and $E_{v}$ denote the embeddings of a category label y and an extended label term v , respectively. The cosine similarity is expressed as (Equation 10):


(10) $$\text{cos }(E_{y},E_{v})=\frac{\sum_{i=1}^{g}E_{y}^{i}E_{v}^{i}}{\sqrt{\sum_{i=1}^{g}{\left(E_{y}^{i}\right)}^{2}}\times \sqrt{\sum_{i=1}^{g}{\left(E_{v}^{i}\right)}^{2}}}$$

where g refers to the dimension of the word embedding, while $E_{y}^{i}$ indicates the $i^{th}$ component of the vector $E_{y}$ .

Notably, to ensure relevance, only the top *N* expanded words with the highest cosine similarity to the category label are preserved, while those with low similarity are excluded.

Probability Prediction: Leveraging contextual cues and prompt templates to estimate the likelihood of masked tokens is a crucial technique in refining the verbalizer. This is achieved using a PLM(e.g., BERT), which outputs the probability distribution over potential words filling the [MASK] position.

More concretely, given a prompt template *T*, the model masks certain words in the input and computes *p*(*T*[*MASK*]), the probability distribution over possible replacements. This distribution reflects the strength of association between each candidate word and the target category.

We apply BERT to obtain this distribution and select the top *N* most probable terms to expand the label word set.

Context Information: In order to enrich the label word set and effectively utilize the surrounding context of masked tokens, we propose an expansion strategy based on context windows. Rather than relying on conventional N-gram models, our approach leverages non-autoregressive PLMs like BERT to capture contextual dependencies. Given that BERT cannot directly estimate full-sentence generation probabilities, we address this constraint through the application of a symmetric sliding window approach.

Assuming a window size of c , the context centered around the [MASK] token can be represented as (Equation 11):


(11) $$W=\left\{\ldots w_{-c},\ldots w_{-1},W,W_{1},\ldots W_{c},\ldots \right\}$$

Within this framework, each word $w_{i}$ in the window is sequentially masked and input into the BERT model for calculating the loss associated with predicting the masked word (Equation 12):


(12) $$L\left(w_{i}\right)=-\sum_{v_{i}\in V}1\left\{v_{i}=w_{i}\right\}\times \text{log }p(v_{i}=w_{i}|W_{w_{i}})$$

where V denotes the vocabulary set, 1 is the indicator function, and $p(v_{i}=w_{i}|W_{w_{i}})$ represents the predicted probability distribution of BERT conditioned on W with $w_{i}$ excluded.

In the experiments, label word candidates are sorted according to their sequence loss *L*(*W*), and those with higher loss values are discarded. Only the words with the lowest losses are preserved. A fixed window size of c=5 is used, and for consistency, the N=15 identified by each of the three strategies are selected to construct the expanded label word set.

The combination of FastText Similarity, Probability Prediction, and Context Information enables a multi311 faceted enhancement of the verbalizer, thereby substantially improving the model’s semantic understanding of category labels.

### 3.5 Final detection

Once the external knowledge-based verbalizer has been refined using the three proposed strategies, we compute the prediction score using a weighted average of the label word scores. In particular, the final prediction $\hat{y}$ is obtained by aggregating the scores of all candidate categories according to their respective word weights. These weights are calculated based on the contribution of each word, as formulated below (Equation 13):


(13) $$\text{arg }\underset{y\in Y}{\max}\frac{1}{\left|V\right|}\sum_{v\in V}p\left(\left[\text{MASK}\right]=v|x_{en}\right)$$

Here, V refers to the collection of label words linked to the category y , while $\left|V_{y}\right|$ indicates the size of this set. The probability function $p(\left[\text{MASK}\right]=\text{v}|{\text{x}}_{\text{en}})$ evaluates how likely the label word v is, conditioned on the enhanced description $x_{en}$ .

## 4 Experiments

### 4.1 Data setting

In this study, we use two benchmark English datasets: the PlantWild dataset and the GojiPest dataset.

PlantWild: PlantWild is a large-scale dataset for wild plant disease recognition, covering multiple healthy plant categories and plant disease categories. It contains over 50,000 images, with each plant disease category accompanied by rich textual descriptions. In this study, we primarily use the textual descriptions from this dataset, which provide detailed explanations of the fine-grained features of various plant diseases, helping the model identify subtle differences between them.

GojiPest: GojiPest is a cross-modal image-text dataset focused on goji plant pests and diseases. It supports tasks such as image collection, text creation, data augmentation, classification, and image-text pairing. The dataset includes images and textual descriptions for various common goji pests and diseases. Similar to the PlantWild dataset, we only use the textual descriptions from this dataset in our study, focusing on utilizing the descriptive information for enhancing the understanding and classification of goji plant pests and diseases.

### 4.2 Baseline methods

In order to assess the effectiveness of our approach, we conducted comparisons with SOTA methods.

Stacked Denoising Autoencoders (SDA) (Zhu et al., 2019): SDA is a conventional unsupervised deep learning model that generates detailed feature representations for both the training and test datasets via an autoencoder. Subsequently, a classifier is trained on labeled data from the training set to carry out classification tasks on the test data.

TextCNN (Kim, 2014): A deep learning network that incorporates a convolutional layer to extract contextual features from labeled training data. Once trained, the model is applied to execute classification tasks on the test set.

BERT (Devlin et al., 2018): BERT (Bidirectional Encoder Representations from Transformers) is based on the Transformer framework. It reformulates tasks into cloze-style (fill-in-the-blank) questions, making it a robust baseline approach for a variety of NLP tasks.

AgriBERT (Chen et al., 2024): AgriBERT is a pre-trained language model specifically designed for agricultural domain texts. It is trained on a large corpus of agricultural literature and technical reports, making it more adept at understanding agricultural terminologies and contexts compared to general-domain PLMs like BERT.

Prompt Learning (PL) (Liu et al., 2021): This method integrates input data from each chunk into a pre-designed template, using only the category name to build the verbalizer in regular prompt-tuning. For consistency, the templates in PL align with those used in our experiments.

P-tuning (Liu et al., 2022): P-tuning is a method for soft prompt-tuning that involves learning continuous prompts by embedding trainable variables into the input representations, instead of using hand-crafted templates.

Mistral (Jiang et al., 2023): Mistral is an emerging large-scale language model created by the Mistral AI team. It is particularly known for its high computational efficiency and robust generative capabilities, outperforming similar models, especially in multimodal tasks.

LLaMA3 (Touvron et al., 2023): LLaMA3 is an efficient, large-scale language model developed by Meta, designed to function well in low-resource environments. It reduces the number of parameters to minimize computational costs while maintaining solid performance in natural language reasoning and generation tasks.

SimSTC (Liu et al., 2025b): A straightforward framework for graph contrastive learning applied to short text classification. The method performs graph learning on various component graphs related to text, generating multi-view text embeddings, upon which contrastive learning is directly applied.

### 4.3 Experiment settings

In this experiment, to generate vague descriptions, we constructed an “expert-non-expert” parallel corpus and fine-tuned a large language model using LoRA technology, enabling it to convert specialized terms into more accessible expressions. Specifically, we first collected approximately 1,200 expert-level symptom descriptions from agricultural manuals and online agricultural knowledge bases. Each description was then paraphrased by GPT-4 into a farmer-style colloquial version, resulting in an expert–non-expert pair. Based on this corpus, we fine-tuned LLaMA-8B with LoRA, using a configuration of rank = 8, *α* = 16, learning rate = 2e-4, and 5 training epochs on dual NVIDIA A6000 GPUs. To ensure quality, we manually filtered the generated sentences to remove incomplete or duplicate expressions. Furthermore, three agricultural experts validated a random sample of 300 pairs, achieving an agreement rate above 90%. This process ensured that the constructed parallel corpus is reliable and suitable for subsequent fuzzification.

We then used the fine-tuned model to apply fuzzification to the text in the dataset. Next, we applied our method to enhance the fuzzified text, resulting in the final dataset for subsequent experiments. The dataset was separated into subsets for training, testing, and validation, with 70% of the data assigned to training, 20% designated for testing, and the remaining 10% kept for validation.

For methods based on deep neural networks and fine-tuned pre-trained language models (e.g., SDA, TextCNN, SimSTC, BERT and AgriBERT), the full training dataset was utilized, as these models necessitate large quantities of data for effective learning. Furthermore, we applied the hyperparameters specified in the original papers to maintain consistency and achieve optimal performance. For the prompt-tuning approaches (PL and P-tuning), a 20-shot configuration was implemented for both methods. To ensure fairness, we kept the parameter settings identical across these approaches: dropout rate was set to 0.5, learning rate to 3e-5, batch size to 32, and weight decay to 1e-5. The hyperparameters, including batch size and learning rate, were determined through repeated empirical experiments to achieve optimal performance. The models were trained for 5 epochs to guarantee thorough training and stable outcomes, with the Adam optimizer employed for parameter tuning. For large language models (e.g., LLaMA3 and Mistral), classification was performed directly by formulating prompts in a question-answer format. Unlike traditional models, these systems do not rely on conventional training techniques but instead fine-tune the prompts specifically for classification tasks.

The effectiveness of our methods is evaluated using the following four key metrics: As Equations 14–17 is shown below.

Accuracy (Acc): The proportion of correctly predicted samples compared to the total number of samples.


(14) $$\text{Accuracy}=\frac{TP+TN}{TP+TN+FP+FN}$$

Precision (Pre): The ratio of positive samples among the predicted positive samples.


(15) $$\text{Precision}=\frac{TP}{TP+FP}$$

Recall (Rec): The ratio of correctly predicted positive samples to the total number of actual positive samples.


(16) $$\text{Recall}=\frac{TP}{TP+FN}$$

F1 Score (F1-S): The harmonic mean of Precision and Recall, used as a comprehensive measure of classification performance. A higher F1 score indicates better overall performance.


(17) $$\text{F}1\text{ Score}=\frac{2\cdot \text{Precision}\cdot \text{Recall}}{\text{Precision}+\text{Recall}}$$

All experiments were conducted on a server equipped with an NVIDIA A100 GPU, a 64-core AMD EPYC 7763 processor, and 512 GB of memory. The experiments were performed using Python 3.9.16 and PyTorch 1.12.0 with CUDA support.

### 4.4 Main results

**Table 2** presents the performance results of our method and baseline models across two datasets (PlantWild and GojiPest). Based on these experimental results, the following insights have been observed:

**Table 2:** The experimental results on three datasets using four different evaluation metrics.

Datasets	Task	Metrics	Methods									
			SDA	TextCNN	BERT	AgriBERT	PL	P-tuning	Mistral	LLaMA3	SimSTC	Ours
PlantWild	Apple	Acc	66.67	71.15	60.79	82.69	80.25	81.73	81.73	82.69	87.89	**95.19**
		Pre	65.02	59.92	63.17	83.81	79.63	81.25	87.85	82.72	87.65	**95.97**
		Rec	66.67	71.15	60.79	84.81	80.79	81.43	73.97	76.80	87.89	**95.20**
		F1	65.83	63.16	60.80	81.76	80.04	80.70	80.31	79.65	87.31	**95.32**
	Corn	Acc	68.91	65.55	55.21	74.79	59.49	61.35	67.23	71.43	80.00	**80.67**
		Pre	69.04	52.32	56.95	77.26	59.30	60.01	80.26	79.45	79.26	**82.78**
		Rec	68.91	65.55	55.21	74.89	60.98	61.67	67.50	71.67	78.00	**80.83**
		F1	68.97	57.33	55.27	75.11	50.67	59.50	73.32	75.36	76.29	**80.66**
	Cucumber	Acc	66.95	79.66	58.34	68.64	63.28	66.95	**88.14**	84.75	78.87	83.05
		Pre	68.44	88.15	61.38	81.01	62.97	66.20	**91.64**	90.63	78.83	82.82
		Rec	66.95	79.66	58.34	68.33	65.39	67.13	**87.93**	84.48	79.87	82.99
		F1	67.69	75.96	60.97	66.50	63.47	65.95	**89.75**	87.45	80.60	82.66
	Tomato	Acc	65.55	72.27	53.53	70.59	58.95	61.26	68.91	57.98	72.50	**73.11**
		Pre	67.64	**81.80**	51.93	72.13	58.21	67.27	67.05	72.69	75.00	73.06
		Rec	65.55	72.27	53.53	70.57	60.09	61.15	68.99	57.70	72.50	**73.30**
		F1	66.57	64.01	52.82	68.60	58.14	64.06	68.01	64.34	71.33	**72.74**
GojiPest	Insect1	Acc	71.91	75.26	59.36	75.11	82.44	90.08	64.96	60.23	81.90	**95.11**
		Pre	79.14	75.53	60.12	73.88	82.58	84.91	73.94	65.78	71.20	**90.61**
		Rec	71.91	75.26	59.36	79.31	85.71	89.71	66.15	68.32	81.90	**96.14**
		F1	75.35	74.68	59.87	75.64	82.45	86.12	70.03	67.03	71.02	**91.31**
	Insect2	Acc	74.83	74.50	55.82	72.28	84.05	90.65	66.78	67.02	92.01	**94.95**
		Pre	75.45	75.46	59.65	73.46	84.50	90.44	59.86	67.52	92.65	**93.94**
		Rec	74.83	74.50	55.82	74.31	85.29	91.04	54.05	68.30	92.01	**94.96**
		F1	75.14	74.67	57.36	73.88	83.94	89.78	56.81	67.91	91.41	**92.95**

The bolder ones mean better.

**Figure 2:**
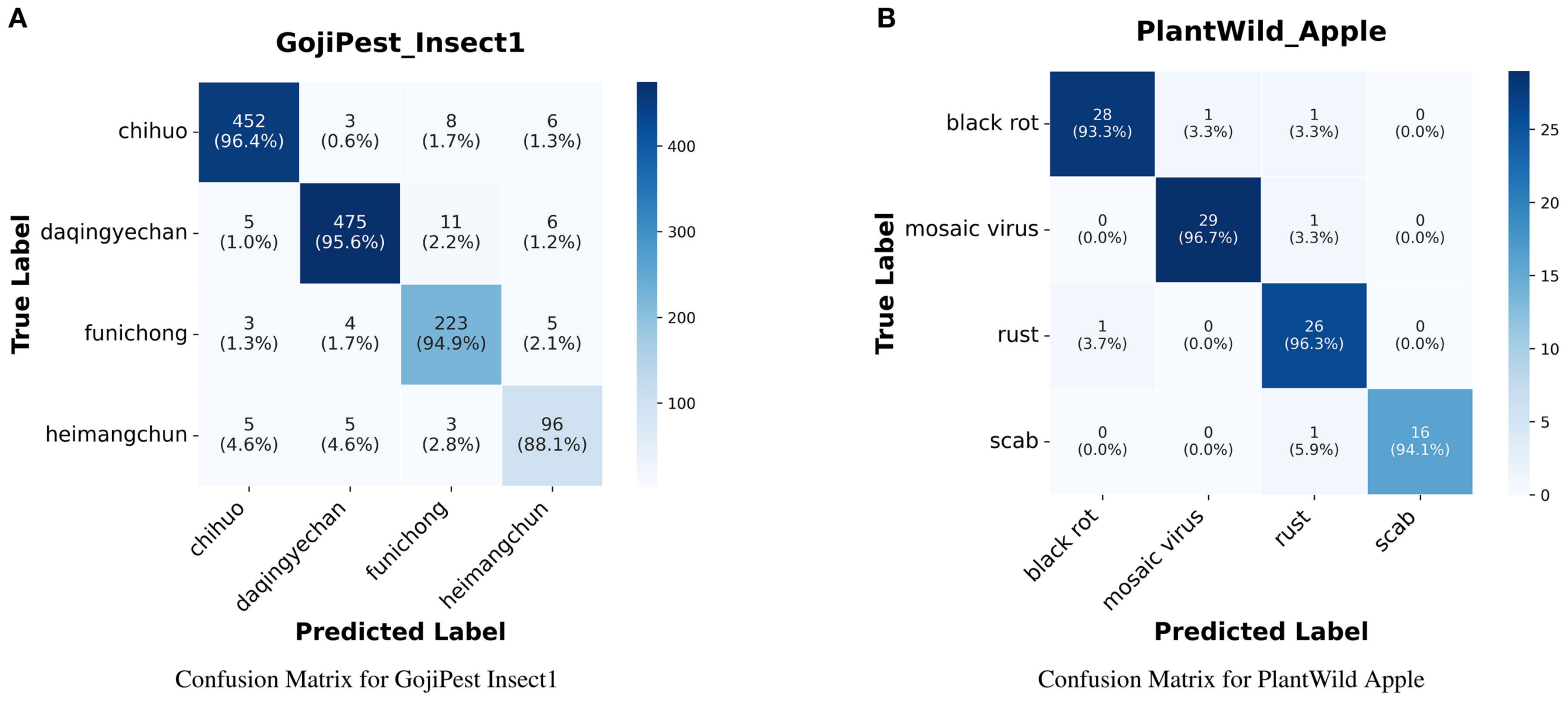
**(A)** shows the confusion matrix for the GojiPest Insect1 dataset and **(B)** shows the confusion matrix for the PlantWild Apple dataset.

1. Our approach consistently surpasses all baseline models across various evaluation metrics. Specifically, for the Apple subtask, it attains much higher accuracy and F1 score, showcasing the effectiveness of our method in few-shot learning scenarios. This result suggests that our method is highly effective at leveraging limited labeled data to achieve superior classification performance compared to other models.
2. Although we used a 15-shot learning setup, which inherently limits the model’s performance due to the small number of training samples, our results show that prompt-based learning significantly improves performance in few-shot scenarios. When compared to traditional deep learning methods like TextCNN and SDA, our approach achieves higher accuracy and F1 scores across multiple subtasks, thus confirming the advantages of prompt learning in handling few-shot tasks.
3. Pre-trained language models like BERT and AgriBERT perform well on agricultural texts but face real-world limitations. Our soft prompt-tuning with a knowledge-enhanced verbalizer outperforms them on colloquial datasets. It effectively handles non-standardized farmer descriptions. Unlike full fine tuning, it updates fewer parameters. This yields higher efficiency and better transferability in few-shot or resource-limited settings.
4. While LLMs such as LLaMA3 and Mistral are powerful, their performance is less stable when applied to short-text tasks, such as plant disease and pest description classification. These models show variability when handling noisy or perturbed inputs, leading to fluctuations in performance metrics. In contrast, our method demonstrates consistent and robust performance, particularly in tasks involving ambiguous or noisy descriptions, underscoring the stability and reliability of our approach.
5. When compared to traditional deep learning models, prompt-based learning models outperform them, especially in the Cucumber subtask, where our method significantly surpassed TextCNN. This suggests that prompt-based learning is better suited to the challenges posed by few-shot learning, allowing the model to more effectively process short-text descriptions and achieve higher classification accuracy.
6. The observed performance differences across the various tasks indicate that prompt-based learning models are adaptable to a range of task characteristics. In particular, our method excels in tasks that involve more complex or detailed descriptions, further demonstrating its ability to generalize effectively across diverse inputs.
7. Visualized confusion matrices from representative subsets further corroborate our method’s effectiveness. On GojiPest Insect1, the model achieved relatively high per-class accuracy, with “chihuo” and “daqingyechan” reaching 96.4% and 95.6%, respectively. On PlantWild Apple, the model cleanly separates disease types with all classes above 93% (e.g., “black rot” 93.3%, “mosaic virus” 96.7%). These results confirm that our approach achieves uniformly high discrimination and robust generalization across categories, as shown in the **Figure 2**.

In conclusion, our method consistently outperforms baseline models across all subtasks, especially in few-shot learning and in handling noisy short-text descriptions. This highlights the advantages of prompt-based learning and knowledge expansion. Future work could explore integrating domain-specific knowledge to optimize prompt templates further and improve the model’s generalization abilities for real-world applications.

### 4.5 The comparative study of large models

To comprehensively evaluate the robustness of our proposed method, we conducted additional experiments along three dimensions: comparisons with fine-tuned large language models, parameter-efficient fine-tuning, and advanced prompting strategies. Specifically, we systematically compared our text-based approach with fine-tuned Qwen, LLaMA with LoRA-based fine-tuning, and Qwen variants equipped with Chain-of-Thought (CoT) reasoning and Self-Consistency (SC) voting under the same few-shot setting.

The experimental results, as shown in **Table 3**, reveal three major findings: (1) Directly using LLMs to analyze agricultural text lacks in-depth reasoning, resulting in performance inferior to methods that incorporate CoT and SC; (2) In scenarios with limited data, LoRA struggles to fully capture the diversity and complexity of the task, making it difficult to generalize effectively to different agricultural text scenarios. This lack of sufficient data support during fine-tuning leads to unstable model performance, with significant drops in certain tasks. (3) Advanced prompting strategies (CoT + SC) bring improvements in some scenarios, but their overall performance is unstable and remains lower than the experimental results of our method.

**Table 3:** Performance comparison of five large language model methods across six tasks.

Datasets	Task	Metrics	Methods				
			Qwen	QwenCoT	Qwen-SC	LLaMALoRA	Ours
PlantWild	Apple	Acc	92.23	85.44	75.96	77.12	**95.19**
		Pre	75.85	71.20	73.70	76.89	**95.97**
		Rec	72.97	66.29	75.96	76.77	**95.20**
		F1	74.38	68.66	74.66	76.83	**95.32**
	Corn	Acc	74.58	74.75	72.44	77.82	**80.67**
		Pre	**86.26**	75.21	72.94	66.51	**82.78**
		Rec	75.00	75.00	72.44	62.66	**80.83**
		F1	80.24	75.11	72.38	64.52	**80.66**
	Cucumber	Acc	79.49	**88.03**	73.90	77.29	**83.05**
		Pre	64.29	72.88	76.99	80.58	**82.82**
		Rec	63.47	70.34	73.90	77.07	**82.99**
		F1	63.87	71.59	70.84	78.79	**82.66**
	Tomato	Acc	66.27	69.66	70.67	66.39	**73.11**
		Pre	76.48	60.89	**79.06**	69.80	**73.06**
		Rec	66.61	53.95	70.67	66.26	**73.30**
GojiPest		F1	71.25	57.24	67.34	67.99	**72.74**
	Insect1	Acc	73.44	74.39	70.44	62.52	**95.11**
		Pre	83.14	58.19	65.61	63.36	**90.61**
		Rec	74.20	63.16	70.44	62.42	**96.14**
		F1	78.41	60.57	64.52	62.88	**91.31**
	Insect2	Acc	72.88	77.91	74.93	62.94	**94.95**
		Pre	73.81	75.21	74.47	68.00	**93.94**
		Rec	53.00	46.24	74.93	60.99	**94.96**
		F1	61.70	57.27	68.99	69.42	**92.95**

Accuracy (%), Precision (%), Recall (%), and F1 (%) are reported.

In addition, an in-depth error analysis shows that LLMs tend to underperform on datasets containing short, ambiguous, and highly colloquial expressions, such as the Tomato dataset. This inconsistency can be attributed to its sensitivity to informal expressions and its limited ability to generalize across heterogeneous agricultural data. In contrast, our method leverages knowledge-enhanced soft prompt-tuning to explicitly bridge colloquial farmer descriptions with formal agricultural terminology, thereby achieving more stable and reliable performance even under noisy and diverse input conditions.

### 4.6 Ablation study

#### 4.6.1 Ablation study on the verbalizer

To accurately evaluate the contribution of the external knowledge-enhanced verbalizer, we conducted ablation experiments to systematically analyze the impact of different construction strategies on model performance. The experiment compared four configurations: a baseline model using only category names as label words, a simple knowledge enhancement method based on synonym expansion, ablated variants employing knowledge graph retrieval but with one of the optimization strategies removed (including FastText similarity filtering, context information ranking, or probability prediction), and the full model integrating all three optimization strategies.

The experimental results, as shown in **Table 4**, demonstrate three key findings: (1) External knowledge is essential, with our full method significantly outperforming the category-name-only baseline (e.g., improving accuracy from 71.15% to 95.19% in the Apple task); (2) Structured knowledge from knowledge graphs proves substantially more effective than simple synonym expansion, which often introduces noise; (3) All three optimization strategies—FastText similarity, probability prediction, and context information—are necessary, as ablating any consistently degrades performance, confirming their joint role in noise filtering and high-quality label word selection.

**Table 4:** The ablation study results across two datasets using four different evaluation metrics.

Datasets	Task	Metrics	Methods					
			Label.	Synonym	-Prob.	-FastText.	-Context.	Full(Ours)
PlantWild	Apple	Acc	71.15	81.73	86.54	85.58	88.46	**95.19**
		Pre	69.84	82.63	85.54	84.53	87.66	**95.97**
		Rec	70.33	81.69	86.41	84.86	88.54	**95.20**
		F1	69.18	80.13	85.71	84.27	87.99	**95.32**
	Corn	Acc	71.43	72.27	75.63	73.11	78.15	**80.67**
		Pre	72.69	76.19	75.52	72.54	78.02	**82.78**
		Rec	71.61	72.47	75.80	73.25	78.30	**80.83**
		F1	71.70	68.28	74.43	71.75	77.90	**80.66**
	Cucumber	Acc	74.58	77.12	77.97	80.51	78.81	**83.05**
		Pre	75.67	78.31	78.41	80.40	79.55	**82.82**
		Rec	74.43	76.87	77.82	80.37	78.59	**82.99**
		F1	74.59	75.32	77.67	79.92	78.59	**82.66**
	Tomato	Acc	61.34	65.55	71.43	70.59	66.39	**73.11**
		Pre	59.68	65.27	73.15	72.00	67.79	**73.06**
		Rec	61.47	65.66	71.52	70.78	66.64	**73.30**
		F1	59.90	64.09	71.70	70.70	63.16	**72.74**
Insect Pest	Insect1	Acc	61.45	71.68	83.89	80.69	81.83	**95.11**
		Pre	56.41	67.39	79.26	75.68	76.72	**90.61**
		Rec	58.07	72.97	85.25	81.69	82.12	**96.14**
		F1	55.42	68.00	81.21	76.94	78.30	**91.31**
	Insect2	Acc	63.98	72.32	80.67	80.47	83.94	**94.95**
		Pre	64.08	75.52	81.54	80.90	84.25	**93.94**
		Rec	62.98	73.61	81.08	81.23	83.66	**94.96**
		F1	62.62	72.69	80.75	80.58	83.80	**92.95**

Bold values indicate the best performance. Label. (Original Label Words), Synonym (Synonym Expansion), -Prob. (without Probability Prediction), -FastText. (without FastText Similarity), -Context. (without Context Information).

**Figure 3:**
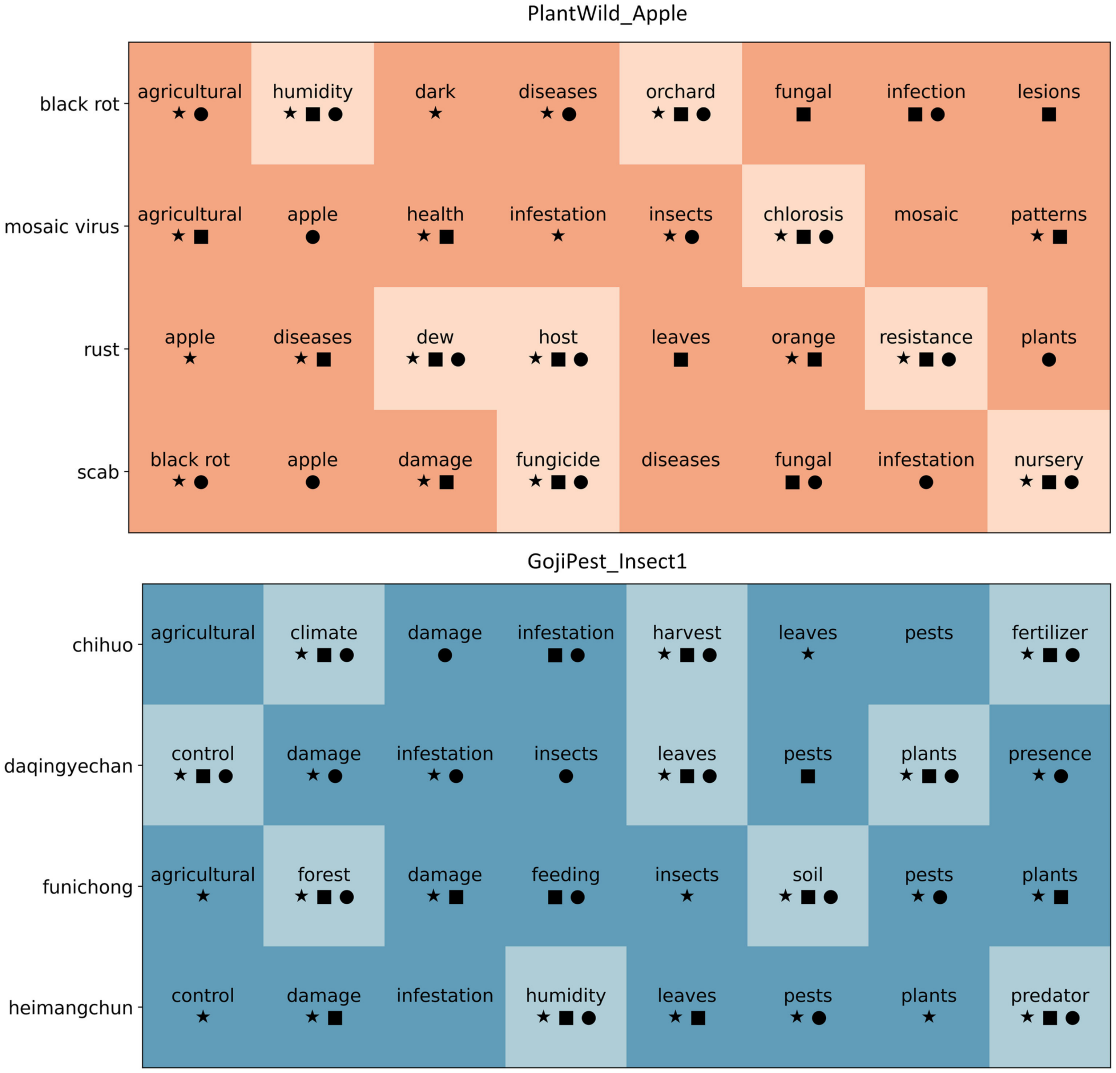
Visualization of the knowledge-enhanced verbalizer on PlantWild Apple and GojiPest Insect1.

To further illustrate the effectiveness of the knowledge-enhanced verbalizer, we provide a visualization of the label word sets associated with different categories under the proposed optimization strategies. **Figure 3** presents representative results from two datasets (PlantWild Apple and GojiPest Insect1). Each row corresponds to a category label, and the words in the row denote the candidate verbalizers. The symbols, which are denoted as square, circle, and star, represent filtering decisions made by removing one of the strategies, namely probability prediction (-Prob.), FastText similarity (-FastText.), and context information (-Context.), respectively. Since we take the union of the three strategies, a word is filtered out only when it is removed by all three, which results in the light-colored blocks, while the dark-colored blocks indicate the retained high-quality label words. As shown in the figure, the full model effectively preserves more informative and semantically relevant label words, while noisy or irrelevant words are gradually removed through the joint optimization strategies. This visualization provides clear evidence of how the proposed verbalizer construction improves both the richness and quality of label word sets, thereby enhancing the overall model performance.

#### 4.6.2 Influence of input data description types

To complement the architectural ablation study and to validate the practical motivation of our work, we further compare the impact of different types of input data on model performance, specifically examining “Original”, “Vague”, and “Expand” (our method) datasets. The “Original” refers to the accurate and detailed descriptions from the dataset, while the “Vague” consists of intentionally blurred text, simulating descriptions that might be provided by non-experts. The “Expand” represents the vague descriptions that 501 are further processed using our method, which leverages prompt-based learning to enhance and refine the 502 textual information, improving its accuracy and specificity.

The experimental results, as presented in **Table 5**, demonstrate that the model exhibits a general decline in performance across all tasks when vague descriptions are used. For instance, in the Corn task, the accuracy drops from 79.83% (Original) to 69.75% (Vague), reflecting the negative impact of vague descriptions on model performance. Similarly, in the Apple and Tomato tasks, the use of vague descriptions results in a noticeable decline in performance. This suggests that vague text introduces additional uncertainty, limiting the model’s ability to classify accurately.

**Table 5:** The results for different types of data across two datasets.

Datasets	Task		Metrics	Methods		
				Original	Vague	Expand(Ours)
PlantWild	Apple		Acc	88.08	80.46	**95.19**
			Pre	88.44	89.69	**95.97**
			Rec	87.06	87.90	**95.20**
			F1	87.74	88.43	**95.32**
	Corn		Acc	79.83	69.75	**80.67**
			Pre	81.99	71.60	**82.78**
			Rec	80.00	69.68	**80.83**
			F1	78.47	69.72	**80.66**
	Cucumber		Acc	79.62	74.58	**83.05**
			Pre	78.64	74.55	**82.82**
			Rec	80.09	74.60	**82.99**
			F1	79.36	74.28	**82.66**
	Tomato		Acc	70.67	67.23	**73.11**
			Pre	71.08	71.79	**73.06**
			Rec	70.83	67.44	**73.30**
			F1	70.95	67.58	**72.74**
Insect Pest	Insect1		Acc	87.86	72.44	**95.11**
			Pre	85.77	72.69	**90.61**
			Rec	88.31	74.84	**96.14**
			F1	87.02	73.75	**91.31**
	Insect2		Acc	86.08	72.97	**94.95**
			Pre	84.76	72.55	**93.94**
			Rec	85.92	73.06	**94.96**
			F1	85.34	72.80	**92.95**

The bolder ones mean better.

However, when “Expand” data is used, where vague descriptions are further processed, the model performance improves significantly. For example, in the Apple task, accuracy increases from 88.08 (Original) to 95.19 (Expand). Similarly, for the Corn, Cucumber, and Tomato tasks, applying our method leads to significant improvements in accuracy. This demonstrates that our expansion method effectively recovers and enhances the information in vague descriptions, improving the model’s handling of such inputs. In some tasks, the performance even surpasses that of the original data. This highlights the efficacy of our approach in enhancing classification performance, particularly in tasks such as pest detection, where ambiguous descriptions pose extra difficulties.

### 4.7 Influence of the templates

In this experiment, the design of templates was pivotal in influencing the model’s performance. To evaluate the effect of various hand-crafted and soft templates on classification tasks related to plant diseaseand pest descriptions, we created and tested several templates, the specifics of which are outlined in **Table 6**. These tasks involve complex short-text descriptions, requiring the model to extract meaningful features and information based on the guidance provided by the templates.

**Table 6:** The different templates on two datasets.

Hard/Soft	id	Template
Manual	0	A {"mask"} condition: {"placeholder": "text-a"}
	1	{"placeholder": "text a"} The type is {"mask"}
	2	{"placeholder": "text-a"} The issue is classified as {"mask"}
	3	{"placeholder": "text a"} A {"mask"} disease
Soft	0	{"soft": "< soft>"}{ "mask": "< mask >"}{ "soft": "< soft>"}{ "placeholder": "text-a"}

“Hard” refers to hand-crafted templates.

The experimental results, displayed in **Table 7**, demonstrate that specific hand-crafted templates successfully direct the model in grasping the essential elements of the tasks, especially in tasks like Insect2, where the model effectively identifies the key features of pest descriptions. However, as the complexity of the tasks and the diversity of the datasets increase, the limitations of using a single, fixed template become evident. For instance, vague or incomplete descriptions may hinder hand-crafted templates from fully leveraging the potential of the data. Consequently, we introduced soft template generation in our approach to improve the model’s ability to process uncertain and ambiguous text. The experimental results demonstrate that, even with limited training data, soft templates can be precisely adapted to the data, thereby creating an optimal theoretical prompt that considerably boosts classification accuracy.

**Table 7:** The 15-shot results of accuracy with different templates on two datasets.

Datasets	Task	Template					Ours
		0	1	2	3	Avg	
PlantWild	Apple	88.46	75.00	89.42	76.92	82.45	**95.19**
	Corn	73.11	66.39	74.79	68.91	70.80	**80.67**
	Cucumber	78.81	72.88	79.66	72.03	75.85	**83.05**
	Tomato	70.59	54.62	63.03	57.98	61.56	**73.11**
Insect Pest	Insect1	92.17	89.54	91.22	84.43	89.34	**95.11**
	Insect2	91.50	85.40	87.02	80.71	86.16	91.31

The bold values indicate the highest accuracy achieved for each task.

### 4.8 Parameter sensitivity

**Figure 4:**
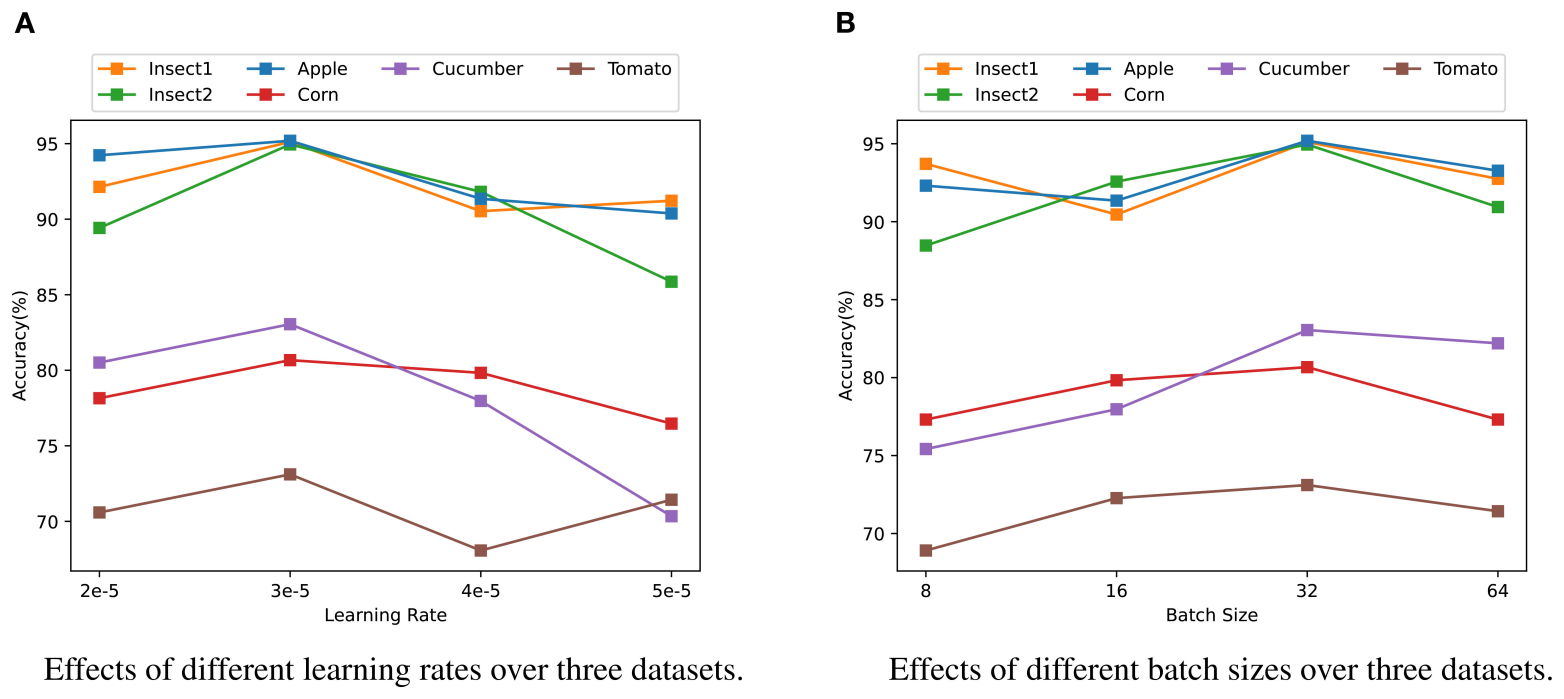
Analysis of model parameter sensitivity on three datasets. **(A)** Effects of different learning rates on the accuracy across three datasets (Insect1, Insect2, Apple, Corn, Cucumber, Tomato). **(B)** Effects of different batch sizes on the accuracy across the same three datasets. Different colors represent different categories: orange for Insect1, green for Insect2, blue for Apple, red for Corn, purple for Cucumber, and brown for Tomato.

We conducted further experiments to evaluate the effect of different hyperparameters, such as learning rate and batch size, on the experimental results. The findings are presented in **Figure 4**. The learning rate governs the magnitude of parameter adjustments during model training. Based on the experimental results, the best performance across most tasks was obtained with a learning rate of 3e-5. This indicates that, within a specific range, a higher learning rate can expedite model convergence and assist the model in adapting to variations in the data. However, for some tasks, setting the learning rate too high can lead to instability during training, emphasizing the importance of fine-tuning the learning rate based on the specific requirements of each task.

In addition to learning rate, batch size is another critical hyper-parameters that influences training dynamics, memory usage, and model convergence. Our findings show that the model performed optimally with a batch size of 32. This suggests that, within an appropriate range, smaller batch sizes can facilitate faster convergence, helping the model adapt quickly to training data, especially for tasks with more complex patterns. On the other hand, larger batch sizes contribute to more stable training and enhanced performance in certain tasks. These results indicate that different datasets and tasks may require distinct batch sizes to achieve the best performance.

Furthermore, we assessed the impact of training epochs on model performance, as shown in **Table 8**.

**Table 8:** The effect of different training epochs on the accuracy of two datasets.

Datasets	Task	Epochs			
		5	10	15	20
PlantWild	Apple	90.38	93.27	95.19	94.23
	Corn	68.07	71.43	80.67	78.99
	Cucumber	73.73	75.42	83.05	75.42
	Tomato	66.39	71.43	73.11	72.27
Insect Pest	Insect1	88.40	92.06	95.11	90.08
	Insect2	86.43	91.37	94.95	88.32

The results indicate that while model performance improves with more epochs, excessive training can lead to diminishing returns. Specifically, in the Apple task from the PlantWild dataset, the model achieved an optimal accuracy of 95.19% after 15 epochs, with a slight decrease to 94.23% at 20 epochs. Similar trends were observed for the Corn and Cucumber tasks, where the best performance was also achieved at 15 epochs, with no significant gain at 20 epochs. These findings suggest that a moderate number of epochs promotes model convergence and generalization, while too many epochs may lead to overfitting. This pattern was further confirmed in the Insect Pest dataset, where 15 epochs also yielded the best results in both Insect1 and Insect2 tasks. In conclusion, selecting an appropriate number of epochs is crucial for optimizing model performance, with 15 epochs proving to be ideal for most tasks.

### 4.9 Validation on a colloquial description dataset

In this section, we constructed a small-sample colloquial description dataset by collecting Q&A pairs from the agricultural platform “Ask Extension^2^” and carefully curating them through filtering and grained annotation. The dataset contains 1,000 samples, covering five categories of plant diseases and pests: Maize Leaf Spot, Rice Blast, Rice Planthopper, and Wheat Powdery Mildew.

On this dataset, we conducted validation experiments using fine-tuned PLMs (AgriBERT), prompt-based learning methods (PL and P-Tuning), the LLM Qwen, as well as our proposed method. The experimental settings and parameters were kept consistent with those in the main experiments. The results are reported in **Table 9**. It can be observed that our method outperforms all other approaches across Accuracy, Precision, Recall, and F1, achieving an F1 Score of 79.70%, which is significantly higher than the second-best method, AgriBERT. In contrast, PL and P-Tuning perform poorly on this small-sample colloquial dataset, indicating the limited generalization capability of traditional prompt-based learning. Although Qwen performs better than the prompt-based learning methods, its adaptation to colloquial data is still insufficient without fine-tuning. Overall, our method demonstrates clear advantages in handling colloquial and standard expressions in classification tasks, providing more accurate and stable predictions across all sample categories.

**Table 9:** Performance of different methods on the newly constructed colloquial dataset.

Metrics	Agribert	PL	P-Tuning	Qwen	Ours
Acc	75.50	63.00	66.00	64.00	79.50
Pre	76.22	56.28	59.21	67.13	80.16
Rec	75.50	63.00	66.00	64.00	79.50
F1	75.17	58.56	61.25	63.62	79.70

## 5 Conclusions and future work

In this paper, we proposed a plant pest and disease classification based on colloquial descriptions by leveraging soft prompt-tuning, which combined AgriBERT-based entity recognition and AgriKG retrieval for knowledge enhancement of input. Then, a soft prompt-tuning method with an external knowledge extension verbalizer is employed for detection. The experimental findings validate that our method outperforms baseline models, including state-of-the-art large language models (LLMs), in detection performance.

In future work, we plan to expand our research in two main directions. Firstly, we will investigate more effective strategies for verbalizer construction, including advanced approaches for generation, filtering, and integration. Secondly, we intend to explore multi-model methods, including computer vision, to derive more robust representations, which can further advance the performance of plant pests and diseases classification. In particular, integrating our model with image description models such as PlanText and leveraging databases like PlantPAD could enable an end-to-end agricultural assistance system that combines descriptive queries with visual observations for more reliable diagnostic recommendations.

## Data Availability

Publicly available datasets were analyzed in this study. This data can be found here: https://www.agridata.cn/data.html#/datadetail?id=289614; https://github.com/tqwei05/MVPDR.

## Footnotes

1
https://relatedwords.org

2
https://ask.extension.org/
